# So Much for Glucosinolates: A Generalist Does Survive and Develop on Brassicas, but at What Cost?

**DOI:** 10.3390/plants10050962

**Published:** 2021-05-12

**Authors:** Verena Jeschke, Jacinta M. Zalucki, Bettina Raguschke, Jonathan Gershenzon, David G. Heckel, Myron P. Zalucki, Daniel G. Vassão

**Affiliations:** 1 Max Planck Institute for Chemical Ecology, 07745 Jena, Germany; verena.jeschke@gmail.com (V.J.); raguschke@ice.mpg.de (B.R.); gershenzon@ice.mpg.de (J.G.); heckel@ice.mpg.de (D.G.H.); 2 Centre for Planetary Health and Food Security, Griffith University, Brisbane 4111, Australia; J.Zalucki@griffith.edu.au; 3 The School of Biological Sciences, The University of Queensland, Brisbane 4072, Australia; m.zalucki@uq.edu.au

**Keywords:** plant chemical defenses, Brassicaceae, glucosinolates, isothiocyanates, generalist herbivore, *Helicoverpa armigera*

## Abstract

While plants produce complex cocktails of chemical defences with different targets and efficacies, the biochemical effects of phytotoxin ingestion are often poorly understood. Here, we examine the physiological and metabolic effects of the ingestion of glucosinolates (GSLs), the frontline chemical defenses of brassicas (crucifers), on the generalist herbivore *Helicoverpa armigera*. We focus on kale and cabbage, two crops with similar foliar GSL concentrations but strikingly different GSL compositions. We observed that larval growth and development were well correlated with the nutritional properties of the insect diets, with low protein contents appearing to exacerbate the negative effects of GSLs on growth, pupation and adult eclosion, parameters that were all delayed upon exposure to GSLs. The different GSLs were metabolized similarly by the insect, indicating that the costs of detoxification via conjugation to glutathione (GSH) were similar on the two plant diets. Nevertheless, larval GSH contents, as well as some major nutritional markers (larval protein, free amino acids, and fat), were differentially affected by the different GSL profiles in the two crops. Therefore, the interplay between GSL and the nitrogen/sulfur nutritional availability of different brassicas strongly influences the effectiveness of these chemical defenses against this generalist herbivore.

## 1. Introduction

Plants of the Brassicaceae constitutively produce glucosinolates (GSLs) as a front line defence against herbivory. Although not toxic per se, when brought together with myrosinase enzymes that are maintained in separate cells/compartments, GSLs are hydrolysed to isothiocyanates (ITCs) and other toxic compounds, detonating the so-called “mustard oil bomb” [1,2,3,4]. The release of these toxic compounds upon plant tissue damage caused by chewing is meant to be a very effective defence against generalist herbivores [5,6,7] and the toxic effects of these compounds have been repeatedly shown in various laboratory assays [8,9,10].

Nevertheless, some 168 species of generalist or polyphagous Lepidoptera across 15 moth families feed on plants in the Brassicaceae, and some 21 are recorded as pests [11]. This includes some key pest insects such as *Spodoptera exigua*, *S. littoralis*, *Mamestra brassicae*, *Trichoplusia ni* and *Helicoverpa armigera*, that were found to metabolize ingested ITCs via conjugation to glutathione (GSH) followed by excretion, suggesting some level of GSL detoxification can play a role when feeding on GSL-defended hosts [12,13]. We attempted to select for improved performance of *H. armigera*, the cotton bollworm, on brassica hosts by exposing neonates to cabbage, and rearing survivors on artificial diet for consecutive generations starting in October 2012. Surprisingly, we found very little effect of our selection on various performance assays: early larval weight gain, time to pupation, pupal weight, time in pupal stage and survival when assessed after 2, 21, and 29 generations of selection [11]. Indeed, both selected and unselected lines produced pupal weights that would be acceptable in assays, although not as “good” as on the routinely used and nutrient-rich artificial diet; see [14] for the relevance of artificial diets. The most consistent effect on performance was that deriving from the larval diet, with brassica plants having strong negative effects on insect development compared with an artificial diet, and with insects fed on cabbage performing less well than those feeding on kale [11], regardless of the number of ancestral generations fed on brassica plants.

Here we determine if there is a biochemical cost for *H. armigera* feeding on brassicas (common cabbage and kale) differing markedly in GSL profiles (see below). We assessed the usual performance measures of herbivorous insects on plant material: larval weight gain, time to complete developmental stages, pupal weight and adult emergence. We also assessed the nutritional chemistry of host processing and tolerance by measuring GSH, free protein and amino acid contents of larvae, and adult fat and protein contents. Given the major biochemical mechanisms previously described for dealing with common GSL-derived ITCs [12,15], we expected that active and continuous detoxification of cabbage and kale ITCs with GSH would have direct negative effects on cellular GSH levels, with further indirect consequences for protein and amino acid levels (especially cysteine and methionine) in larvae, as well as on the body composition, such as fat levels, in adults.

## 2. Results

### 2.1. Diet and Food Plant Composition

**Figure 1:**
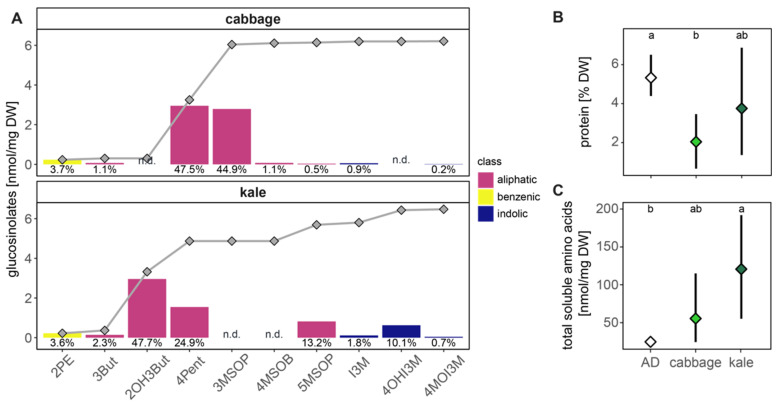
Glucosinolate (**A**), protein (**B**) and soluble amino acid content (**C**) of the food plants and artificial diet (AD). (**A**) Absolute amounts of single glucosinolates [nmol/mg DW] detected in cabbage (*n* = 6) and kale (*n* = 5) are plotted as bars, the colour refers to the class of the glucosinolate: benzenic, aliphatic or indolic. The contribution of that structure to the total glucosinolate amount is printed as percentage below the corresponding bar. The dots represent the accumulated means of glucosinolates. (**B**) Soluble protein [% DW] of the diets (*p* = 0.031) and (**C**) total soluble amino acids [nmol/mg DW] (*p* = 0.011) plotted as mean ± 95% confidence interval. Tukey letter denoted a statistical difference of 0.05. Mean, standard deviations and statistical details for all analytes are in Table S1. AD = artificial, diet, DW = dry weight, n.d = not detected; glucosinolate side chains: 2PE = 2-phenylethyl, 3But = 3-butenyl, 2OH3But = 2-hydroxy-3-butenyl, 4Pent = 4-pentenyl, 3MSOP = 3-(methylsulfinyl)propyl, 4MSOB = 4-(methylsulfinyl)butyl, 5MSOP = 5-(methylsulfinyl)pentyl, I3M = indol-3-ylmethyl, 4OHI3M = 4-hydroxy-indol-3-ylmethyl, 4MOI3M = 4-methoxyindol-3-ylmethyl.

Samples of artificial diet, kale and cabbage were analysed for their glucosinolate content (plant samples), total protein, soluble amino acids and water content to compare overall nutritional and toxin load. Both cabbage (6.21 ± 2.62 nmol/mg DW, *n* = 6) and kale (6.47 ± 3.58 nmol/mg DW, *n* = 5) have comparable total GSL levels (Welch’ s t-test, *p* = 0.954, df = 7.68) but differ in their composition (Figure 1A; Figure S1), as well as in protein levels (Figure 1B) and total soluble amino acid (Figure 1C). The two dominating GSL in cabbage were 4-pentenyl- (4pent) and 3-methylsulfinylpropyl (3MSOP)- GSL (accounting for 92%) and in kale 2-hydroxy-3-butenyl- (2OH3But) and 4pent-GSL were the most abundant (accounting for 73%, Figure 1A). In both plants, the GSL profile was dominated by aliphatic-derived GSL (95% for cabbage, 84% for kale) with lower contributions from indolic (1% for cabbage, 12% for kale) and benzenic GSLs (4% for cabbage, 3% for kale; Figure S1). Soluble protein content was lower in plant samples (2.0% DW in cabbage and 3.8% DW in kale) compared to the artificial diet (5.3% DW, ANOVA, *p* = 0.031; Figure 1B). In contrast, soluble free amino acids were significantly higher in cabbage (55.49 nmol/mg DW) and kale (120.71 nmol/mg DW) compared to artificial diet (24.71 nmol/mg DW, ANOVA, *p* = 0.011; Figure 1C). Relative amounts of individual amino acids also differ between both plant samples (Table S1) and generally it is observed that there is a high variance in the detected amino acids levels across all measured samples. Lastly, all diet samples have comparable water content; 84% for artificial diet (variance = 3%, *n* = 5), 92% (variance = 1%, *n* = 8) for cabbage and 91% (variance = 1%, *n* = 10) for kale; plant diets did not differ, (*p* > 0.05, t-test) but both had higher water content that the artificial diet (*p* < 0.05).

### 2.2. Caterpillar Development and Body Chemistry

The effects of different GSL-containing diets on the development of the two *H. armigera* strains were assessed comparing survival, larval weight at 4–5 days after hatching (III instar) and their body chemistry (protein content, GSH level and amino acid levels). We did not find any significant effect of the long-term rearing (selection) of *H. armigera* on cabbage or kale in the larval developmental or chemical responses to the offered diet, thus “strain” was omitted in the analysis and only included as a random factor (nested ANOVA with mixed effect model). Survival of larvae at 4–5 days after hatching (larval stage III) was best on diet, next on kale and worst on cabbage (see [11]). The higher mortalities during the larval stage on cabbage and kale may be due to handling, as their diets were changed every 1–2 days, whereas diet-reared larvae were relatively undisturbed. Mortality was high during the pupal stage for both kale and cabbage reared larvae. Overall survival to the adult stage was 74% on diet, 45% on kale and 28% on cabbage [11].

**Figure 2:**
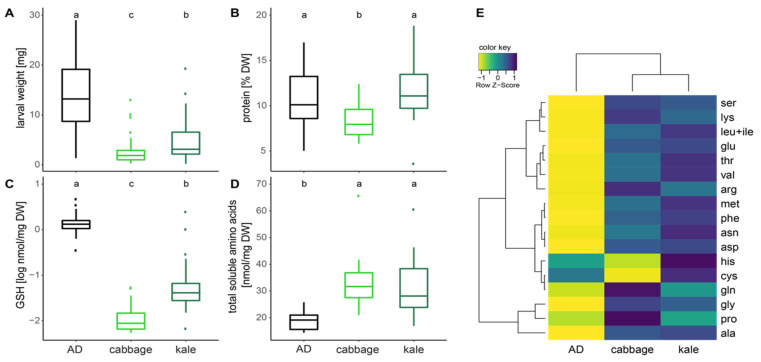
Larval weight and body nutritional composition when fed on artificial diet and food plants with different GSL contents. (**A**) Larval fresh weight at 4–5 days (III instar) in mg (*p* < 0.001, *n* = 80–152); Figure S2 depicts the corresponding values for dry weight. (**B**) Protein in % of dry weight (*p* = 0.001, *n* = 20); (**C**) GSH concentrations in log of nmol/mg dry weight (*p* < 0.001, *n* = 20); (**D**) Total soluble amino acids in nmol/mg dry weight (*p* < 0.001, *n* = 20); (**E**) Heatmap of the mean [nmol/mg DW] of detected soluble amino acids, with yellow to green corresponding to small values and green-blue to dark blue to high values. Data were scaled across rows, comparing amounts of these amino acids between diet treatment groups. Statistical differences for A-D were assessed using a nested ANOVA mixed affect model, letters denote statistical difference at *p* = 0.05 level tested with Tukey post hoc test. Means, standard deviations and statistical details for A-D and individual amino acids (**E**) are in Table S2. AD, artificial diet; DW, dry weight; ▪ indicate outliers in the box-and-whiskers plots.

Larval growth (weight gain) was fastest on artificial diet, and larvae fed with cabbage and kale were differently affected and grew significantly smaller (*p* < 0.001, Figure 2A). Kale-fed caterpillars have similar protein levels as those on control diet; in contrast, cabbage-fed caterpillars, which also showed the strongest growth reduction, had significantly lower protein levels (*p* = 0.001, Figure 2B). Similarly, the measured decrease of intracellular GSH levels was strongest for cabbage, where levels were reduced to 1% of those of the control group, but kale-fed caterpillars also had significantly lower GSH-levels (14% compared to the control group; *p* < 0.001, Figure 2C). Caterpillar dry weight shows the same pattern as GSH levels (Figure S2), which suggests growth may be coupled via nutrition to GSH metabolism. Both plant-fed caterpillar groups show an increase in total free soluble amino acids (*p* < 0.001, Figure 2D), which suggests an increased body protein degradation. Most individual free amino acids showed increased levels up to two-fold in plant-fed caterpillars (Figure 2E, Table S2), but there were notable exceptions. Absolute levels of cysteine were lowered in cabbage-fed caterpillar, but with a high variance across the population. Cabbage-fed caterpillars also showed low levels of histidine, but high levels of glutamic acid, proline, and serine. In contrast, histidine levels were elevated in kale-fed caterpillars. Of particular interest are the amino acids building GSH, namely cysteine (and its potential in vivo precursor methionine), glutamic acid and glycine. Relative levels of those amino acids (% of total amino acids) showed interesting patterns (Figure S3). Cysteine contributes the lowest proportion in plant-fed caterpillars, with cabbage-fed caterpillars being most strongly affected (*p* < 0.001). Relative levels of glutamic acid show a similar pattern (*p* < 0.001, while those of glycine are inversely increased (*p* = 0.039). Notably, relative levels of methionine are elevated in plant-fed caterpillars; however, levels are reduced in cabbage-fed caterpillars compared to kale-fed caterpillars (*p* = 0.017). Overall, cabbage-fed caterpillars are more strongly affected in larval fresh and dry weight, protein levels, GSH levels and individual amino acid levels than kale-fed caterpillars.

**Figure 3:**
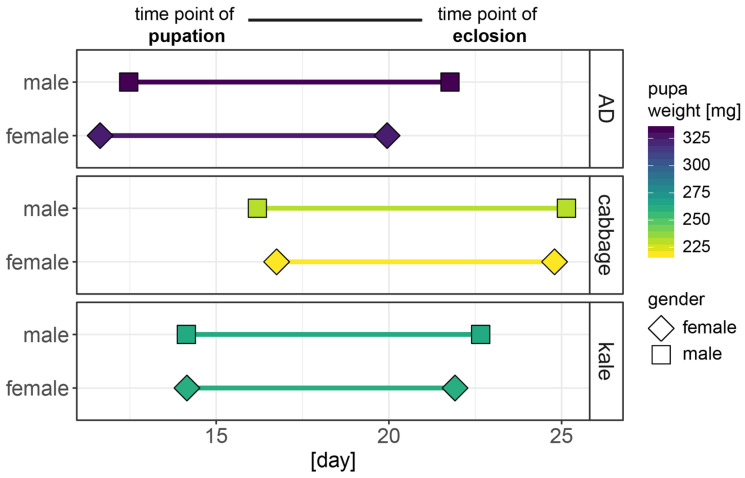
Time to pupation or completion of the larval stage (left data point) and time to adult eclosion (right data point) in days since hatching. The line represents the overall time as a pupa, the colour highlights the pupal weight (taken 24 h after start of pupation). Males are depicted as boxes, females as diamonds, *n* = 44–92. Means ± SE and statistical information can be found in Figure S4 and Table S3.

The drastic effects caused by the Brassica diets on larval weight and body chemistry further influence overall caterpillar development time, time in the pupal stage and pupal weight (Figure 3). Caterpillars exposed to GSLs spend more time in the larval stage than caterpillars fed on artificial diet (time point of pupation, Figure 3), with cabbage-fed caterpillars being more affected than kale-fed caterpillars (*p*(diet) < 0.001, *n* = 44–92). On average, larvae fed artificial diet entered the pupal stage at 12 days after hatching, while the larval stage was prolonged for kale-fed caterpillars for another two days and cabbage-fed caterpillars for another 4–5 days (Figure S4A, Table S3). There is no significant effect on gender alone on the time point of pupation, but a diet-gender interaction; that is, cabbage-fed females enter pupation later than the males, while diet-fed females pupated earlier than the males (*p*(diet×gender) = 0.003, Figure 3).

Pupal weight showed an inverse pattern to the pupation time points: pupae resulting from larvae fed artificial diet were significantly heavier than those reared on plants (*p*(diet) < 0.001, Figure 3). Pupae from the cabbage group were 30% lighter, while pupae from the kale group were 20% lighter than those of the control group (Figure S4C, Table S3). There was no significant effect of gender on pupal weight (Table S3), but cabbage-fed females were noticeably lighter than their male counterparts. 

Similar to the time point of pupation, the time point of eclosion was significantly delayed for the plant-fed individuals (Figure 3). Interestingly, the duration of the pupal stage was significantly shorter for insects of the cabbage- and kale-treatment groups by 4% and 8%, respectively, compared to the control groups (*p*(diet) < 0.001, Table S3). Males from the kale group had the strongest reduction of pupal time with a decrease of 0.8 days compared to the control group, while females from the kale group had the overall shortest time of pupation. Overall, females have significantly shorter duration of pupation than males (*p*(gender) < 0.001). Consequently, females had significantly shorter durations for overall development until eclosion across all diets (Figure 3, *p*(gender) < 0.001). Despite the quicker pupal development for the plant groups, cabbage- and kale-fed adults emerged significantly later than those of the control groups (*p*(diet) < 0.001, Figure S4). This resulted in an average shift of eclosion of 4.9 days for cabbage-treated females and 3.3 days for cabbage-treated males, while kale-treated females and males emerged only 2 days and 1 day later compared to the control group (Table S3). 

### 2.3. Adult Body Weight and Composition

**Figure 4:**
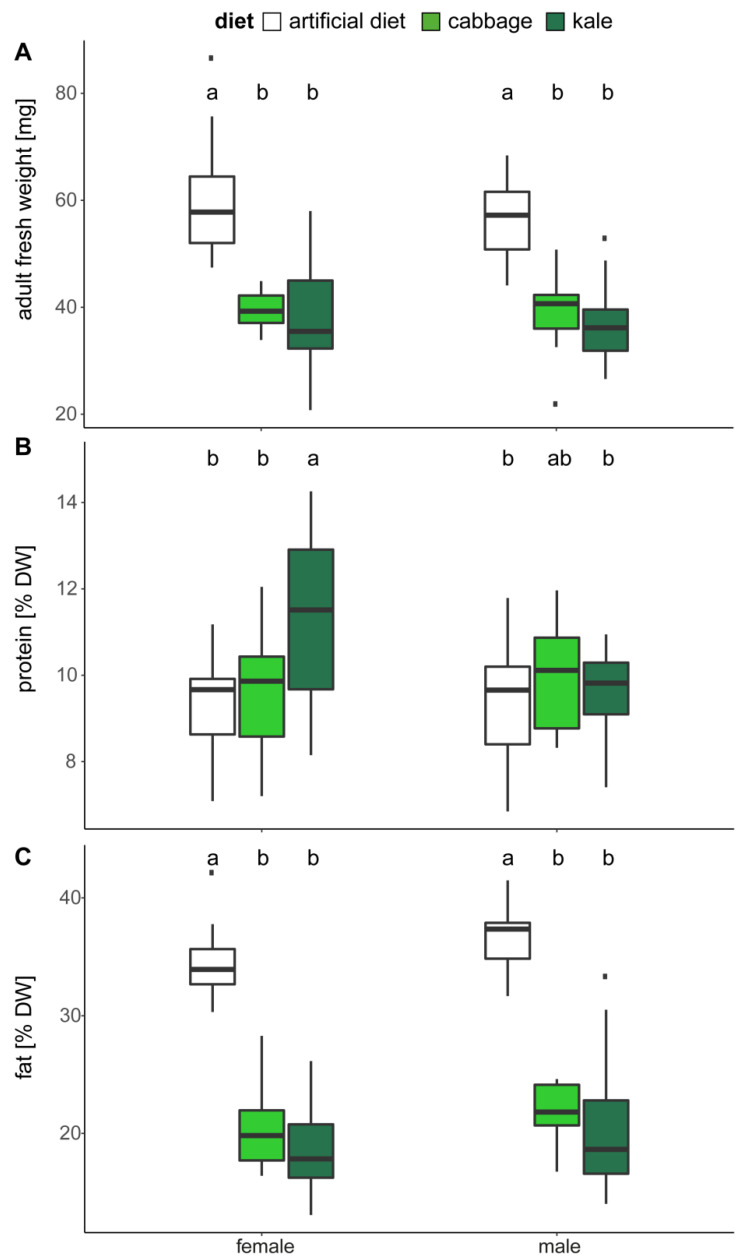
Adult body composition after eclosion. (**A**) Adult fresh weight is strongly affected by the larval diet (*p*(diet) < 0.001, *p*(gender) = 0.286, *p*(diet×gender) = 0.752). (**B**) The amount of protein (per dry weight) is differentially affected by diet for females and males (*p*(diet) = 0.002, *p*(gender) = 0.033, *p*(diet×gender) = 0.008). (**C**) The amount of fat (per dry weight) is mainly affected by larval diet (*p*(diet) < 0.001, *p*(gender) = 0.041, *p*(diet×gender) = 0.885). Mean, standard deviation and statistical details are in Table S4. Statistical differences were assessed using nested ANOVA with mixed effect model, letters denote statistical differences at the *p* = 0.05 level tested with Tukey posthoc test; ▪ indicate outliers in in the box-and-whiskers plots.

Similar to their differences in pupal weights, adults reared on cabbage or kale had lower body weight than those reared on artificial diet (*p*(diet) < 0.001, Figure 4A), but weights were not affected by gender within a diet type. In contrast, protein levels in male and female adults were differently affected by cabbage or kale feeding (Figure 4B, *p*(diet) = 0.002, *p*(gender) = 0.033, *p*(diet*gender) = 0.008). Females raised on kale had the highest levels of protein, while protein levels were elevated in males raised on cabbage. Body fat mirrored the pattern of fresh adult body weight with adults reared on diet having highest fat content (28.8%/DW), followed by kale (17.7%/DW) and cabbage (13%/DW; *p*(diet) < 0.001, Figure 4C). Females from all treatment groups had slightly lower fat levels than the males (*p*(gender) = 0.041).

### 2.4. Conjugate Excretion in Faeces

**Figure 5:**
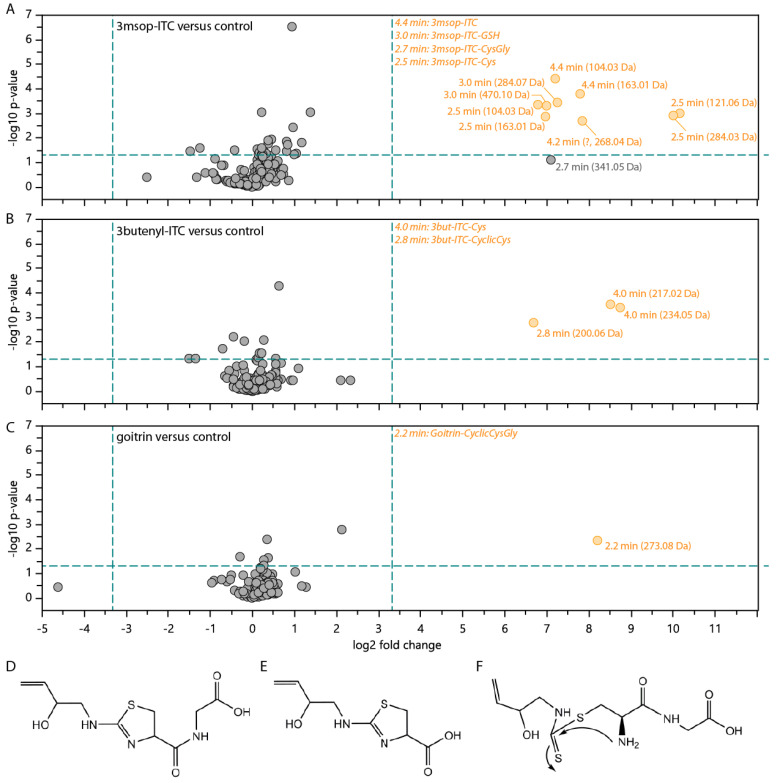
Glucosinolate hydrolysis products are metabolized via the mercapturic acid pathway and intramolecular cyclizations by *H. armigera* larvae. (**A**–**C**) Volcano plots of extracted LC-MS/MS features from non-targeted UHPLC-HRMS analyses indicate that the major metabolites of 3msop-ITC (**A**), 3but-ITC (**B**), and goitrin (**C**) are all formed by conjugation to glutathione (GSH), further hydrolysis of the amino acid constituents of GSH to give CysGly and Cys conjugates, and intramolecular cyclizations to give cyclic conjugates. The proposed goitrin metabolite (**D**) corresponds to the MS feature at 2.2 min in (**C**), while a signal corresponding to the proposed metabolite (**E**) was detectable in manually extracted ion chromatograms from faeces extracts but was too small to be extracted automatically by the software. The proposed formation of these cyclic metabolites is shown in (**F**).

In order to determine the differences in metabolism of glucosinolate-derived ITCs and goitrin, we performed a non-targeted UHPLC-HRMS analysis of frass from *H. armigera* larvae fed on artificial diets containing those compounds. Goitrin is the product of an intramolecular rearrangement (cyclization) of the ITC formed by hydrolysis of 2-hydroxy-3-butenyl glucosinolate, and due to the lack of an exposed ITC group, can be expected to undergo different metabolism than ITCs. This non-targeted analysis indicated that very few products were formed by the caterpillar metabolism, i.e., only a few MS signals were significantly different between extracts of frass from toxin- and control-fed larvae (Figure 5). Most of those products could be readily assigned to putative structures, based on comparison of retention times, molecular masses and fragmentation patterns to product mixtures, as the expected mercapturic acid pathway metabolites. In frass from 3msop-ITC-fed larvae (Figure 5A), signals matching 3msop-ITC and its metabolites 3msop-GSH, 3msop-CG and 3msop-Cys accounted for most of the mass features detected. In frass from 3-butenyl-ITC-fed larvae (Figure 5B), the ITC-Cys conjugate was easily detected, while an additional LC-MS feature corresponded to a product of its intramolecular cyclization, as previously reported for 4msob in slugs [16]. The comparison of extracts from faeces of goitrin- and control-fed larvae (Figure 5C), however, only indicated one product as a potential goitrin product, but its mass and molecular formula did not match any of the expected products of the mercapturic acid pathway. Further analysis of its MS fragmentation strongly suggested that this was a product of an intramolecular cyclization reaction similar to that in slugs [16], but deriving from the goitrin-CysGly conjugate (Figure 5D). A corresponding LC-MS peak was present in a synthetic reaction mixture of goitrin and CysGly. Based on this information, the corresponding cyclic product of the putative goitrin-Cys (Figure 5E) conjugate was detected in the crude reaction mixture of goitrin and Cys, as well as in frass extracts, although its intensity was too low in the latter for it to have been picked by the analysis software. Following the proposed cyclization mechanism (Figure 5F), the corresponding products of goitrin-GSH and goitrin-NAC are less favourably formed, as the cysteine amine is less active as a nucleophile. Accordingly, these compounds were not detected in frass, or in reaction mixes of goitrin with GSH and NAC.

## 3. Discussion

Plant specialized and defensive compounds are highly variable among plants, and GSL in Brassica plants are no exception. Since these metabolites are key components of several human vegetable crops, they have been extensively measured, and found to vary greatly in both amount and composition among Brassica crops (e.g., [17]), plant parts (e.g., [18]), among varieties (e.g., [19]) and seasonally (e.g., [20,21]). In the two species studied here, the overall GSL levels were the same but the composition within the group of aliphatic GSLs differed greatly: Cabbage contained mostly 3-methylsulfinylpropyl (3msop)-GSL (absent from kale), and kale 2-hydroxy-3-butenyl- (2OH3But)-GSL (progoitrin, absent from cabbage). These aliphatic GSLs will both generate ITCs upon hydrolysis, with the product of 2OH3But-GSL undergoing further intramolecular rearrangement/cyclization to form goitrin. Post-feeding factors therefore help to modulate toxicity and larval performance.

The proposed insecticide-like toxicity of isothiocyanates (ITC) [22] is believed to derive at least partially from the reaction of the electrophilic ITC group (–N=C=S) with the intracellular tripeptide nucleophile glutathione (GSH), resulting in its depletion and instigating other metabolic consequences [12]. Conjugation with GSH is a common post-ingestion metabolic pathway for mitigating the toxicity of glucosinolate-derived ITCs in generalist lepidopteran herbivores feeding on *Arabidopsis*, including *H. armigera* [13]. The full GSH-ITC conjugate and its downstream CysGly- and Cys conjugates were the major ITC-derived metabolites produced by the generalist *S. littoralis* [12,13]. 

The balance between conjugation and dissociation reactions is a dynamic process, which can eventually release the individual amino acid constituents of GSH as well as free ITC, which can conjugate with GSH again, leading to depletion of the intracellular GSH pool (Equation 1):

(1) $$\mathrm{ITC}\;\overset{+\mathrm{GSH}}{\to }\mathrm{ITC}-\mathrm{GSH}\overset{-\mathrm{Glu}}{\to }\mathrm{ITC}-\mathrm{CysGly}\;\overset{-\mathrm{Gly}}{\to }\mathrm{ITC}-\mathrm{Cys}$$

Equation (1) describes larval detoxification process in gut after ITC ingestion, where ITC-GSH, ITC-CysGly, ITC-Cys represent conjugates of ITCs with glutathione, cysteinylglycine, and cysteine respectively.

To maintain homeostatic levels of free GSH while it is being heavily used in detoxification processes, the insect needs to consistently synthesize more GSH. High protein diets can help replenish the necessary amino acids, particularly cysteine which is the limiting substrate in GSH biosynthesis. A high-protein diet can reduce the influence of potential toxins on the development of *H. armigera* [23]. In this study, we found that kale had a higher protein and soluble free amino acid content than cabbage (Figure 1B,C). Concurrently, cabbage-fed larvae grew more slowly than kale-fed larvae (lower weight at age 4–5 days, Figure 2A), had lower levels of protein (Figure 2B) and GSH (Figure 2C) and took longer to enter pupation and to complete overall development to adulthood (Figure 3). These different physiological effects between cabbage- and kale-fed insects were present despite them having been exposed to similar GSL levels (Figure 1A), and of the major GSL hydrolysis products in each plant (4-pentenyl- and 3-methylsulfinylpropyl-ITCs in cabbage, and goitrin, a rearranged product without the exposed –N=C=S group of ITCs in kale) being presumably metabolized via the same mercapturic acid pathway (Figure 5). This strongly suggests that the nutritional indices of the two host plants, especially their nitrogen and sulphur amino acid contents and bioavailabilities, strongly affect the toxicity of their chemical defences.

While kale-fed larvae contain about 10% of the amounts of GSH of control larvae fed on artificial diet, the GSH levels of cabbage-fed larvae were yet 10-fold lower compared to those of the kale-fed group (Table S2). This suggests a strong depletion of GSH used in combating free ITCs and other oxidative stresses upon feeding on Brassicaceae. Yet, we find significantly increased absolute levels of free glutamic acid (Table S2) suggesting that glutamic acid is not a limiting amino acid; however, relative amounts of Glu (percentage of total amino acids) decrease in plant-fed larvae suggesting an increased usage for GSH synthesis (Figure S3) and/or enlarged free amino acid pools resulting from body protein catabolism. Similar to what had been seen in a previous study [12], cysteine levels were also depleted in *H. armigera* larvae feeding on GSL-containing diets (Figure S3). Due to the relative toxicity of this sulphur-containing amino acid [24], free cysteine makes up only a small proportion of total free amino acids, about 0.3% for the control larvae and even less in Brassicaceae-fed larvae. It is suggested that in lepidopteran caterpillars more than 20% of the insect´s total cysteine is allocated to GSH [25], making this an invaluable and limiting substance. Hence, the limitations in cysteine availability consequently limit GSH for detoxification; however, critical minimal levels must be maintained for a balanced metabolism to ensure redox homeostasis, on the one hand, and the formation of new proteins for growth or enzymes for essential bioreactions, on the other. That is, lepidopteran larvae can dynamically redirect some of their protein and amino acid resources towards replenishing GSH via an increased availability of its constituent amino acids, especially cysteine. The resulting lower protein levels in larvae exposed to ITCs may be a consequence of an active protein degradation to free up critical amino acids (Figure 2D, Table S2) and energy for detoxification, or from lowered protein synthesis due to limitations in certain amino acids [12]. Here, larvae feeding on cabbage, which produces the aliphatic methylsulfoxide-containing 3msob-GSL (Figure 1), strongly depleted cysteine for use in GSH biosynthesis and resulted in an imbalanced protein metabolism (Figure 2) and thus slower larval growth, in agreement with our previous findings [12]. Interestingly, this effect was also found in parasitoids of *Plutella xylostella* larvae that were raised on GSL-rich *A. thaliana* Col-0 [26]. A recent study, in which *H. armigera* larvae were fed with sinigrin (allyl GSL), confirms that protein metabolism is disturbed in GSL-fed larvae, with upregulations in transcripts related to glutathione and amino acid metabolism [27]. Inversely, adults have elevated protein levels, but significantly lower fat for both genders (Figure 4). This suggests extensive consequences of GSL toxicity on overall resource usage not only during larval development, but also during metamorphosis. The insect fat bodies serve as a central energy storage to power movement and flight and play crucial roles in hormonal regulation and crucially vitellogenesis, an essential step in egg formation and reproduction [28].

Furthermore, we found that individuals raised on GSL-containing plants had significantly longer developmental times for all stages (Figure 3). Again, this effect was stronger for individuals on the cabbage diet (containing the aliphatic 3msop-GSL), and delays in pupations have previously been seen in *S. littoralis* larvae raised on *A. thaliana* Col-0, which contains largely methylsulfoxide-aliphatic GSLs like 4msob and 3msop, among others [29]. Similarly, parasitoids developing on *P. xylostella* larvae that fed on 4msob-ITC-infused leaves suffered from delayed development, lower adult emergence success, and lower body fat content [26] further indicating that GSL-derived compounds affect general animal metabolism, and that these effects are not unique to lepidopteran herbivores. 

The prolonged developmental duration and delayed pupation and adult eclosion may have several detrimental implications for the species fitness. A reduced larval growth rate, which has been demonstrated for aliphatic and indolic GSLs affecting different lepidopteran species [7,12,29,30], is detrimental in a natural setting due to longer exposure to predators (slow growth-high mortality hypothesis) and longer times competing for food resources. Another important consequence of late eclosion comes with the overall fitness cost of producing fewer generations per season [31,32]. Interestingly, *S. littoralis* that were raised on cabbage as larvae were shown to avoid Brassicaceae for oviposition [33,34] suggesting behavioural modification to ensure the best chances of survival for the offspring and species. 

In conclusion, we show that the lifelong exposure to GSLs and their derived hydrolysis products detrimentally affects insect development and alters body chemistry in the generalist herbivore *H. armigera*. The ingestion of these chemicals led to a depletion of GSH as a consequence of their detoxification via the mercapturic acid pathway, further leading to an imbalanced metabolism of proteins and amino acids in a diet-dependent manner. Somewhat surprisingly, these effects were apparently not ameliorated after continuous rearing of this species on Brassicaceae plants for several generations, suggesting a lack of plasticity of this ancient and basic metabolic pathway used for detoxification and redox homeostasis. Ultimately, the prolonged developmental times and delayed eclosion caused by GSL-derived toxins, in combination with changes in adult metabolism, can influence the number and health of *H. armigera* larvae and render GSL efficient defensive metabolites against generalist herbivores.

## 4. Materials and Methods

### 4.1. Plants

Cabbage (*Brassica oleracea* var capitata, White cabbage “Gloria” (F1 hybrid) from Daehnfeldt Seeds) and kale (*Brassica napus*, Rape broadleaf Essex Salad Sproutin, from B&B World Seeds) were grown in trays (58 cm × 32 cm × 11.5 cm) in a peat-based substrate (Klasmann Kultursubstrat TS1, Geeste-Grob Hesepe) under greenhouse conditions at 21–23 °C, 50–60% RH and 14:10 L:D photoperiod. Each tray contained approximately 60 plants. Multiple trays were established every seven days ensuring similar quality food was available for experiments. Plants were used when they were ca. six weeks old.

### 4.2. Plant Chemistry and Diet Composition

Six leaves were collected from six week-old plants per species, flash-frozen in liquid nitrogen and lyophilized using a freeze-dryer. Water content in the diet was assessed using gravimetric methods. Dried and ground plant material was analysed for GSL content as described in [29], with *p*-hydroxybenzyl GSL (sinalbin) as an internal quantification standard. The contents of protein und soluble amino acids were determined by extracting 10 mg dried and ground plant material in 100 μL aqueous buffer (Tris, 50 mM, pH 7.5). Protein content was quantified using Bradford assay as described in [12] and soluble amino acid content was quantified via LC-MS/MS as described in [35]. 

### 4.3. Insects

The *H. armigera* were from the Toowoomba strain (TWB3) maintained at 28–30 °C on artificial diet purchased from BioServ (Cat. No. F9772, Frenchtown, NJ, USA) and a putative “cabbage feeding” strain we designate as TKF (“Toowoomba Kohlfütterung”). The protocol for maintenance of the insects can be found in [36]. We undertook selection of the TWB3 strain to see if feeding on cabbage in the first instar could select for “better” performance upon GSL exposure. Larvae from isofemale lines were exposed to cabbage leaves in Petri dishes (9 cm diameter) and survivors to the third instar were then reared on artificial diet, mated and the offspring of single pair mating re-exposed to cabbage for each generation starting in October 2012. Subsets of the hatched larvae from each rearing group were used in experiments after 29 generations, in October 2017, as described below.

### 4.4. Experimental Protocol

We set up single pair crosses within TWB3 and TKF strains and collected eggs daily. Newly hatched larvae (0–8 h old) were introduced to cabbage or kale leaves placed on moist filter paper in Petri dishes, or on artificial diet (9 cm diameter, 10 larvae/dish). All experiments were conducted in a 29 °C environment cabinet, 65% RH, 12:12 L:D (Snijders Scientific, model EB2E). 

Larval survival and weight gain were assessed after four to five days; at that temperature larvae were 51–68 degree-days old (about instar III). Larvae used for experiments and sample collections were then individualized into rearing cups (36.9 ml Solo Ultra Clear Portion Containers) with their respective diets and labelled. Subsets of larvae across all families were weighed individually (Mettler XS105 Dual Range balance) before being put into cups. There was sufficient artificial diet in a cup for each larva to feed ad libitum until development was complete. Larvae on kale and cabbage were moved onto fresh leaf material daily till they completed development. The time to pupation was recorded and pupae weighed once the cuticle had hardened (within 24 h). Pupae were checked twice daily, and newly emerged adults sexed.

We collected frass from first instars, late instars and 24 h post molt larvae for each diet treatment and source (TWB3 and TKF, chosen from ca. two larvae per diet per family) for analysis of physiological performance on various diets and targeted metabolite profiling of ITC-derived compounds. These larvae and newly emerged adults (< 24 h old) were collected for profiling of body chemistry and composition. Samples were flash frozen in liquid nitrogen and stored at −80°C until further processing.

### 4.5. Caterpillar and Adult Body Chemistry

Lyophilized and weighed whole caterpillar and adult samples were ground and extracted with 100 μL Tris buffer (50 mM, pH 7.5) per mg dry weight and analysed for total protein levels using Bradford assay, total fat content using a gravimetric method, and GSH and soluble amino acids using LC-MS/MS as described in Jeschke et al. [12]. 

### 4.6. Artificial Diet Feeding and Excretion Assays

Fourth-instar *H. armigera* larvae (*n* = 15) were fed on an artificial diet mix [36] containing 1 mmol/kg of selected glucosinolate hydrolysis products (3-methylsulfinylpropyl-ITC, 3-butenyl-ITC, or goitrin). Larvae were fed in individual 37-mL plastic cups for 2 days, faeces from groups of three larvae (*n* = 5) were collected, and 20–40 mg aliquots were extracted with 0. 5 mL methanol:water (1:1) containing 0.5% formic acid (*v/v*) with vigorous shaking. Solid debris were pelleted by centrifugation, and the supernatants were transferred to new vials and analysed by UHPLC-MS.

### 4.7. Non-Targeted UHPLC-MS Analyses of Larval Frass

Larval faeces extracts were analysed by UHPLC (Thermo Dionex Ultimate 3000) coupled to an HRMS-qTOF MS system (Bruker timsTOF, Bremen, Germany). The UHPLC was equipped with a C18 reversed phase column (Zorbax Eclipse XDB-C18, 1.8 μm, 2.1 mm × 100 mm; Agilent Technologies, Böblingen, Germany) maintained at 25 °C and operated at 0.3 mL/min with a gradient flow of 0.1% aqueous formic acid (solvent A) and acetonitrile (solvent B) with the following profile: 0.5% B from 0–0.5 min, 0.5–80% B from 0.5 to 11 min, 80–100% B from 11–11.1 min and kept at 100% B until 12 min, then re-equilibrated at 0.5% B from 12.1–15 min. HRMS analyses were performed separately at positive and negative ionization modes, with automatic MS2 scans (“autoMS”) enabled. The source end plate offset was kept at 500 V and the capillary voltage at 4500 V, with the nebulizer gas at 1.8 bar, dry gas at 10 L/min and a drying temperature of 230 °C. Ion transfer was performed with a funnel 1 RF of 150 Vpp, funnel 2 RF of 200 Vpp, multipole RF of 50 Vpp and a deflection delta of 70 V, with the quadrupole ion energy maintained at 4 eV (low mass 90 m/z), and a collision energy of 7 eV and pre-pulse storage of 5 us. The mass scan range was 50–1500 m/z at an acquisition rate of 12 Hz. Collision energies were stepped in a 50:50 timing between a collision RF of 400 Vpp with transfer time 62.5 us and 800 Vpp/80 us, respectively. Masses were calibrated with m/z of sodium formate adducts injected at the beginning of each chromatographic analysis. Chemical standards of predicted mercapturic acid products of 3msop-ITC, 3-butenyl-ITC and goitrin for chromatographic and MS/MS comparison were prepared synthetically: 5 mM of the GSL hydrolysis products were mixed with 5 mM of glutathione, cysteinylglycine, cysteine or *N*-acetylcysteine in water: ethanol 1:1 at room temperature for 24–48 h, and used directly for HPLC-MS/MS analyses. Data were analysed using the MetaboScape 4.0 software (Bruker, Bremen, Germany), with extraction of features present in at least 3 samples with peak height >5000. Peaks deemed not present in specific samples were assigned an intensity value of 50 counts for fold-change calculations.

### 4.8. Statistical Analyses

All statistical testing was performed using the statistical software R 3.6.1 (R Development Core Team, http://www.r-project.org, accessed on 7 May 2021). All data were checked for statistical prerequisites such as homogeneity of variances and normality. The tests used are described in the respective figure and table legends. In short, differences in the diet composition were assessed with ANOVA or Wilcoxon test. Caterpillar and adult chemistry and composition were assessed using a nested ANOVA with mixed effect model (*nlme*-package, [37]) with the strain as the random factor and the diet and gender (for adults) as fixed factors. Since the strain did not have a significant influence on any of the analytes investigated in this study it was not included as a fixed factor for the analysis. 

## Data Availability

The data presented in this study are available in the article and in the Supplementary Materials.

## Supplementary Materials

The following are available online at https://www.mdpi.com/article/10.3390/plants10050962/s1, Figure S1: Concentrations of glucosinolates from different biosynthetic classes in cabbage and kale, Figure S2: Dry weights of larvae fed on artificial diet (AD), cabbage, or kale, Figure S3: Relative amounts of selected amino acids in bodies of caterpillars fed on artificial diet (AD), cabbage, or kale, Figure S4: Development of larvae fed on artificial diet (AD), cabbage, or kale, (A) duration of the larval stage, (B) duration of the pupal stage, (C) pupal weights, (D) total developmental time until adult eclosion, Table S1: Protein levels, soluble free amino acid composition and glucosinolate compositions of the diets used, Table S2: Larval weights and composition 4–5 days after hatching, Table S3: Duration of insect development, Table S4: Weights and proportions of protein and fat in adult insects.

## Abbreviations

glucosinolate (GSL), isothiocyanate (ITC), glutathione (GSH), 2-phenylethyl (2PE), 3-butenyl (3But), 2-hydroxy-3-butenyl (2OH3but), 4-pentenyl (4Pent), 3-(methylsulfinyl)propyl (3MSOP), 4-(methylsulfinyl)butyl (4MSOB), 5-(methylsulfinyl)pentyl (5MSOP), indol-3-ylmethyl (I3M), 4-hydroxy-indol-3-ylmethyl (4OHI3M), 4-methoxyindol-3-ylmethyl (4MOI3M).

## References

[1] Burow M., Losansky A., Müller R., Plock A., Kliebenstein D.J., Wittstock U. (2009). The Genetic Basis of Constitutive and Herbivore-Induced ESP-Independent Nitrile Formation in Arabidopsis. Plant Physiol. (Bethesda).

[2] Halkier B.A., Gershenzon J. (2006). Biology and biochemistry of glucosinolates. Ann. Rev. Plant Biol..

[3] Matile P. (1980). The mustard oil bomb. Compartmentation of the myrosinase system. Biochem. Physiol. Pflanz..

[4] Wittstock U., Burow M. (2007). Tipping the Scales—Specifier Proteins in Glucosinolate Hydrolysis. IUBMB Life.

[5] Hopkins R.J., Dam N.M.V., Loon J.J.A.V. (2009). Role of Glucosinolates in Insect-Plant Relationships and Multitrophic Interactions. Ann. Rev. Entomol..

[6] Li Q., Eigenbrode S.D., Stringam G.R., Thiagarajah M.R. (2000). Feeding and Growth of *Plutella xylostella* and *Spodoptera eridania* on *Brassica juncea* with Varying Glucosinolate Concentrations and Myrosinase Activities. J. Chem. Ecol..

[7] Müller R., de Vos M., Sun J.Y., Sønderby I.E., Halkier B.A., Wittstock U., Jander G. (2010). Differential Effects of Indole and Aliphatic Glucosinolates on Lepidopteran Herbivores. J. Chem. Ecol..

[8] Badenes-Perez F.R., Reichelt M., Gershenzon J., Heckel D.G. (2013). Interaction of glucosinolate content of *Arabidopsis thaliana* mutant lines and feeding and oviposition by generalist and specialist lepidopterans. Phytochemistry (Oxford).

[9] Bejai S., Fridborg I., Ekbom B. (2012). Varied response of *Spodoptera littoralis* against *Arabidopsis thaliana* with metabolically engineered glucosinolate profiles. Plant Physiol. Biochem..

[10] Zalucki M.P., Zalucki J.M., Perkins L.E., Schramm K., Vassão D.G., Gershenzon J., Heckel D.G. (2017). A Generalist Herbivore Copes with Specialized Plant Defence: The Effects of Induction and Feeding by *Helicoverpa armigera* (Lepidoptera: Noctuidae) Larvae on Intact *Arabidopsis thaliana* (Brassicales) Plants. J. Chem. Ecol..

[11] Zalucki J.M., Heckel D.G., Wang P., Kuwar S., Vassão D.G., Perkins L.E., Zalucki M.P. (2021). A generalist feeding on Brassica; it does not get any better with selection. Plants.

[12] Jeschke V., Gershenzon J., Vassão D.G. (2016). A mode of action of glucosinolate-derived isothiocyanates: Detoxification depletes glutathione and cysteine levels with ramifications on protein metabolism in *Spodoptera littoralis*. Insect Biochem. Mol. Biol..

[13] Schramm K., Vassão D.G., Reichelt M., Gershenzon J., Wittstock U. (2012). Metabolism of glucosinolate-derived isothiocyanates to glutathione conjugates in generalist lepidopteran herbivores. Insect Biochem. Mol. Biol..

[14] Katsikis C.I., Wang P., Zalucki M.P. (2020). Life history traits of a key agricultural pest, *Helicoverpa armigera* (Lepidoptera: Noctuidae): Are laboratory settings appropriate?. Aust. Entomol..

[15] Gols R., Bukovinszky T., Van Dam N.M., Dicke M., Bullock J.M., Harvey J.A. (2008). Performance of generalist and specialist herbivores and their endoparasitoids differs on cultivated and wild Brassica populations. J. Chem. Ecol..

[16] Falk K.L., Tokuhisa J.G., Gershenzon J. (2007). The Effect of Sulfur Nutrition on Plant Glucosinolate Content: Physiology and Molecular Mechanisms. Plant Biol..

[17] Hwang I.M., Park B., Dang Y.M., Kim S.-Y., Seo H.Y. (2019). Simultaneous direct determination of 15 glucosinolates in eight Brassica species by UHPLC-Q-Orbitrap-MS. Food Chem..

[18] Bhandari S.R., Jo J.S., Lee J.G. (2015). Comparison of glucosinolate profiles in different tissues of nine brassica crops. Molecules.

[19] Sasaki K., Neyazaki M., Shindo K., Ogawa T., Momose M. (2012). Quantitative profiling of glucosinolates by LC–MS analysis reveals several cultivars of cabbage and kale as promising sources of sulforaphane. J. Chromatogr. B Anal. Technol. Biomed. Life Sci..

[20] Cartea M.E., Velasco P., Obregón S., Padilla G., de Haro A. (2008). Seasonal variation in glucosinolate content in Brassica oleracea crops grown in northwestern Spain. Phytochemistry (Oxford).

[21] Choi S.-H., Park S., Lim Y.P., Kim S.-J., Park J.-T., An G. (2014). Metabolite profiles of glucosinolates in cabbage varieties (Brassica oleracea var. capitata) by season, color, and tissue position. Horticult. Environ. Biotechnol..

[22] Wittstock U., Kliebenstein D.J., Lambrix V., Reichelt M., Gershenzon J. (2003). Chapter five Glucosinolate hydrolysis and its impact on generalist and specialist insect herbivores. Recent Adv. Phytochem..

[23] Wang P., Vassao D.G., Raguschke B., Furlong M.J., Zalucki M.P. (2021). Balancing nutrients in a toxic environment: The challenge of eating. Insect Sci..

[24] Williamson J.M., Boettcher B., Meister A. (1982). Intracellular Cysteine Delivery System That Protects against Toxicity by Promoting Glutathione Synthesis. Proc. Natl. Acad. Sci. USA.

[25] Barbehenn R.V., Kochmanski J., Menachem B., Poirier L.M. (2013). Allocation of Cysteine for Glutathione Production in Caterpillars with Different Antioxidant Defense Strategies: A Comparison of Lymantria Dispar and Malacosoma Disstria. Arch. Insect Biochem. Physiol..

[26] Sun R., Gols R., Harvey J.A., Reichelt M., Gershenzon J., Pandit S.S., Vassão D.G. (2020). Detoxification of plant defensive glucosinolates by an herbivorous caterpillar is beneficial to its endoparasitic wasp. Mol. Ecol..

[27] Jagdale S., Tellis M., Barvkar V.T., Joshi R.S. (2021). Glucosinolate induces transcriptomic and metabolic reprogramming in Helicoverpa armigera. 3 Biotech.

[28] Li S., Yu X., Feng Q. (2019). Fat Body Biology in the Last Decade. Ann. Rev. Entomol..

[29] Jeschke V., Kearney E.E., Schramm K., Kunert G., Shekhov A., Gershenzon J., Vassão D.G. (2017). How glucosinolates affect generalist lepidopteran larvae: Growth, development and glucosinolate metabolism. Front. Plant Sci..

[30] Schlaeppi K., Bodenhausen N., Buchala A., Mauch F., Reymond P. (2008). The glutathione-deficient mutant pad2-1 accumulates lower amounts of glucosinolates and is more susceptible to the insect herbivore Spodoptera littoralis. Plant J. Cell Mol. Biol..

[31] Benrey B., Denno R.F. (1997). The slow-growth-high-mortality hypothesis: A test using the cabbage butterfly. Ecology (Durham).

[32] Clancy K.M., Price P.W. (1987). Rapid herbivore growth enhances enemy attack: Sublethal plant defenses remain a paradox. Ecology (Durham).

[33] Proffit M., Khallaf M.A., Carrasco D., Larsson M.C., Anderson P., Dam N. (2015). ‘Do you remember the first time?’ Host plant preference in a moth is modulated by experiences during larval feeding and adult mating. Ecol. Lett..

[34] Thöming G., Larsson M.C., Hansson B.S., Anderson P. (2013). Comparison of plant preference hierarchies of male and female moths and the impact of larval rearing hosts. Ecology (Durham).

[35] Docimo T., Reichelt M., Schneider B., Kai M., Kunert G., Gershenzon J., D’Auria J.C. (2012). The first step in the biosynthesis of cocaine in Erythroxylum coca: The characterization of arginine and ornithine decarboxylases. Plant Mol. Biol..

[36] Celorio-Mancera M.D.L.P., Heckel D.G., Vogel H. (2012). Transcriptional analysis of physiological pathways in a generalist herbivore: Responses to different host plants and plant structures by the cotton bollworm, *Helicoverpa armigera*. Entomol. Exp. Appl..

[37] Pinheiro J., Bates D., DebRoy S., Sarkar D., Team R.C. (2007). Linear and Nonlinear Mixed Effects Models. R Package Version.

