# Supplementary figures and images for: So Much for Glucosinolates: A Generalist Does Survive and Develop on Brassicas, but at What Cost?
Source: Plants (Basel). 2021 May 12;10(5):962. doi: 10.3390/plants10050962 (PMC8150600; doi:10.3390/plants10050962)

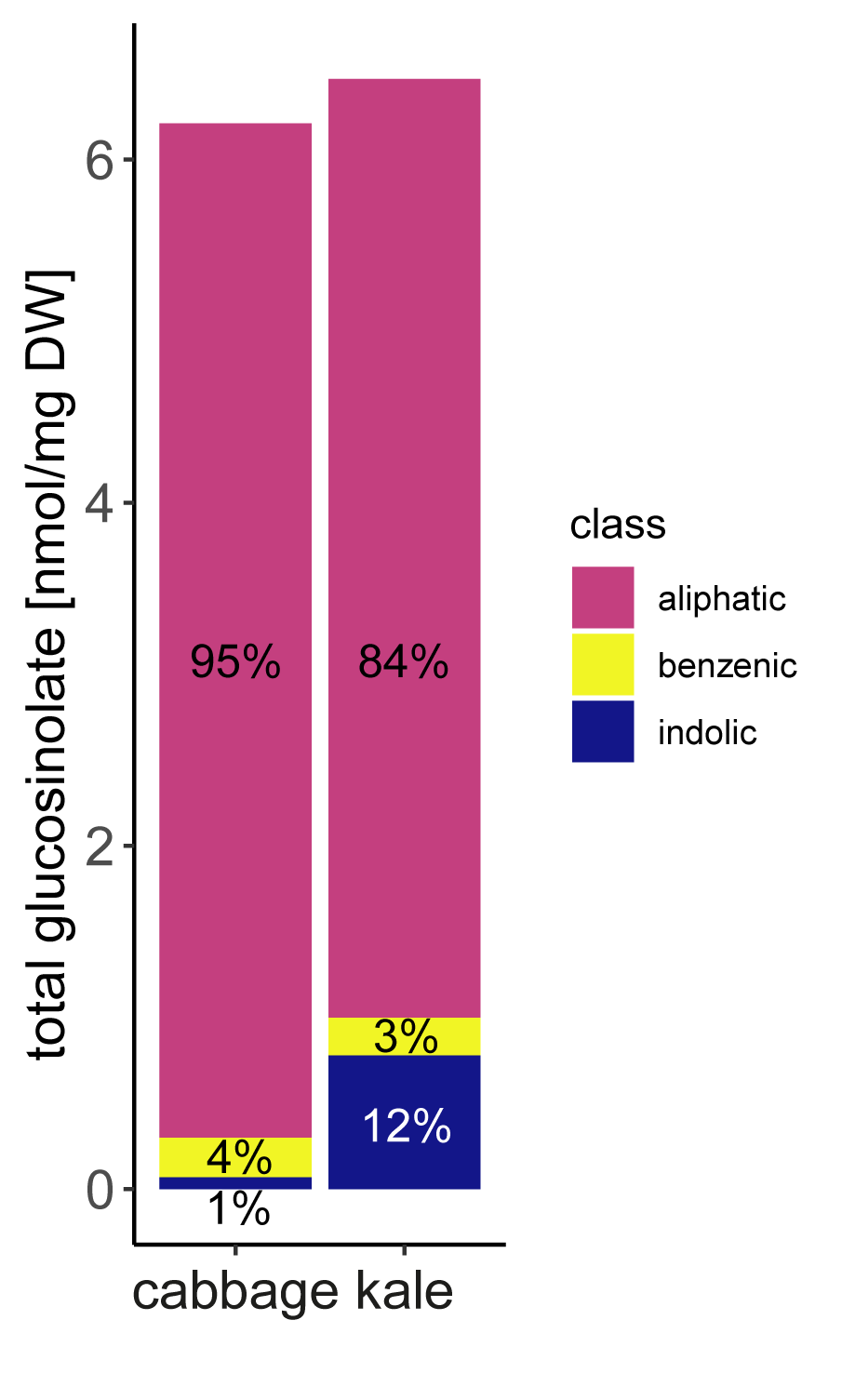

Supplement: Supplementary file 1 [file plants-10-00962-s001.zip › SupplFig1.tif]

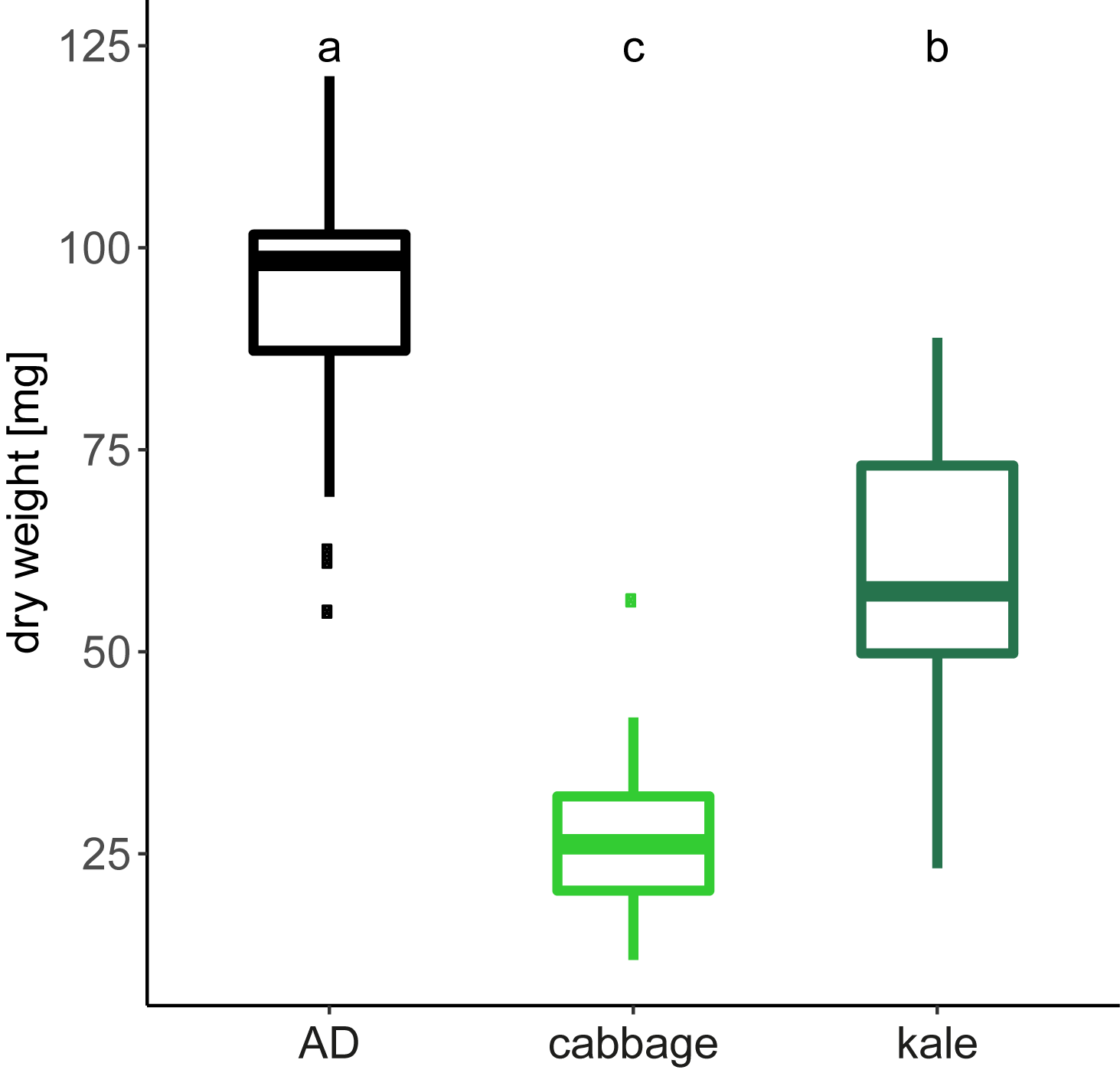

Supplement: Supplementary file 1 [file plants-10-00962-s001.zip › SupplFig2.tif]

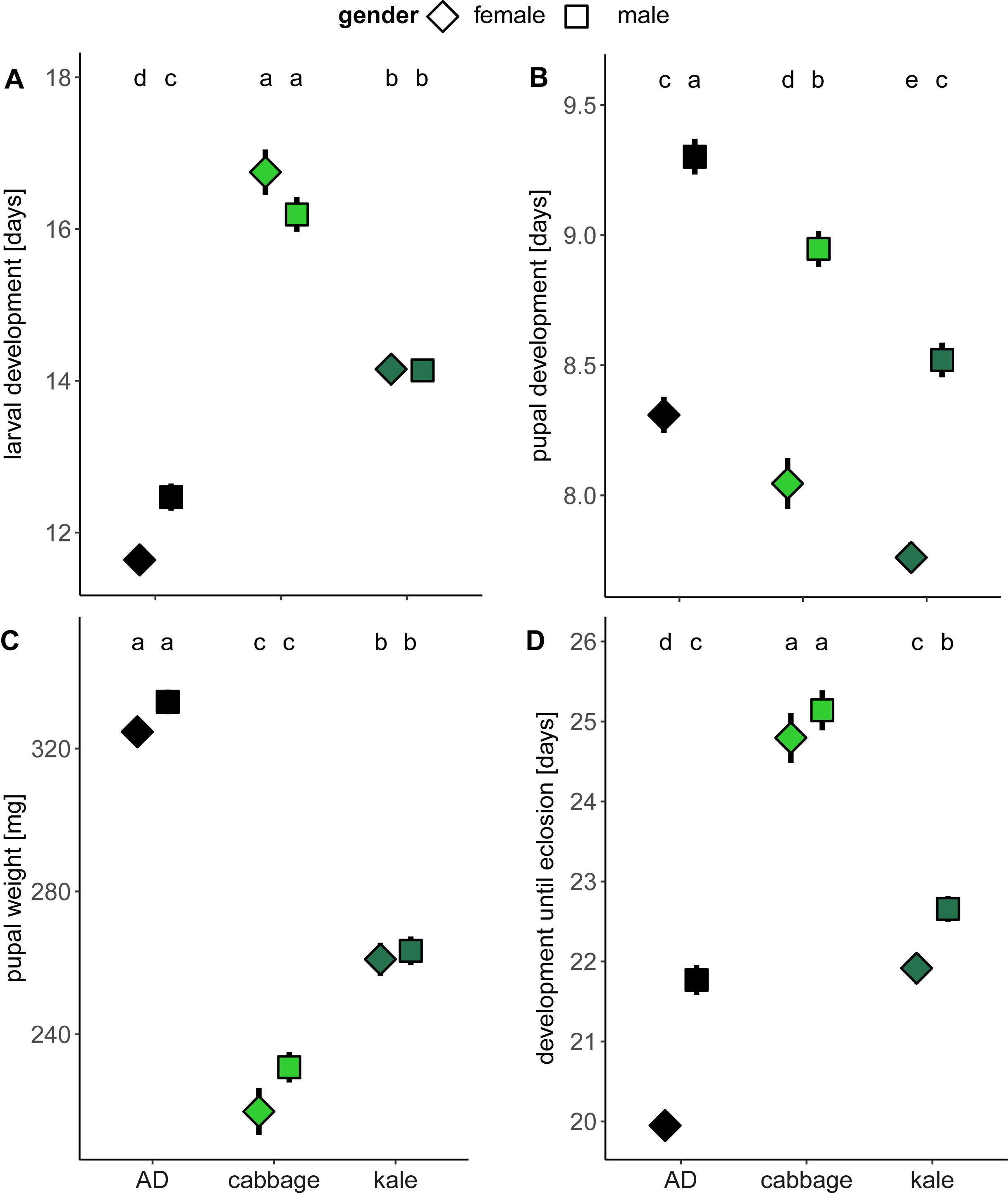

Supplement: Supplementary file 1 [file plants-10-00962-s001.zip › SupplFig4.tif]
